# Case report: Successful treatment of a thallium sulfate intoxication in a dog with use of Prussian blue

**DOI:** 10.3389/fvets.2025.1528327

**Published:** 2025-02-05

**Authors:** Fergal M. McDermott, Anne A. Kan, Claudine C. Hunault, Joris H. Robben, Dylan W. de Lange, Marieke A. Dijkman

**Affiliations:** ^1^Section of Emergency and Intensive Care Medicine, Department of Clinical Sciences, Faculty of Veterinary Medicine, Utrecht University, Utrecht, Netherlands; ^2^Dutch Poisons Information Center, University Medical Center Utrecht, Utrecht, Netherlands

**Keywords:** canine, thallium poisoning, rodenticide, gastrointestinal, alopecia

## Abstract

Soluble thallium salts are highly toxic, with mortality rates exceeding 70% in animals compared to 6%−15% in humans. Early identification of thallium intoxicated patients and decreasing the toxic load by targeted treatment using Prussian Blue are associated with a better outcome in humans. Prussian blue, however, is rarely available or used in veterinary settings. Here we present a rare report of the successful use of Prussian Blue in the management of a dog with a thallium intoxication. A 5-year-old miniature Schnauzer, ingested a thallium sulfate based rodenticide leading to lethargy, anorexia, regurgitation, abdominal pain, borborygmi, constipation, ataxia, dermatitis and progressive alopecia. Intervention involved supportive care with subcutaneous fluids, carprofen, butylscopolamine and dexamethasone in combination with targeted treatment using the chelating agent Prussian blue (15 days) followed by activated charcoal (4 days). The serum thallium concentration on the 5th day of the Prussian Blue treatment was 196 mcg/L confirming thallium intoxication. The serum thallium concentrations were 20.7 mcg/L and 21.5 mcg/L on days 14 and 34 of treatment, respectively. The calculated elimination half-life during the during PB treatment was 2.8 days. The patient showed gradual improvement over several weeks, achieving full recovery at 11 weeks. This case emphasizes the importance of a timely diagnosis and the early start of targeted therapy using Prussian blue, in the management of thallium intoxication in veterinary patients.

## Introduction

Thallium (Tl) is an extremely toxic heavy metal. It is odorless, colorless and tasteless and has been implicated in both intentional and accidental intoxications (1). Because of its high toxicity, the World Health Organization recommended against its use as rodenticide in 1973, and many countries have since restricted its use (2). Nevertheless, old Tl-based rodenticides are still available in private storage and Tl is still used in semiconductors, artificial jewelery and certain laboratory growth media. Therefore, human and veterinary Tl intoxications are still being reported (3–5).

Thallium salts are readily absorbed through the gastrointestinal tract, the respiratory tract and the skin and the absorption after ingestion may be prolonged in case of Tl-induced paralytic ileus. Thallium has a wide range of toxicological effects on cellular function. Due to its chemical resemblance to potassium (e.g., similar charge and ionic radius) it can replace potassium thereby disrupting various vital potassium-dependent processes such as the functioning of Na+/K+ ATPase pumps (4–7). In the rabbit kidney, Tl has a demonstrated affinity 10 times greater than potassium for Na+/K+ ATPase pumps (3, 6). The sulfhydryl groups in proteins and mitochondrial membranes are also effected by Tl, leading to the disruption of oxidative phosphorylation by the inhibition of enzymes like pyruvate kinase and aldehyde dehydrogenase, leading to mitochondrial damage and disrupted calcium homeostasis (4, 6). Reactive oxygen species are also generated by Tl through the disruption of the mitochondrial electron transport chain, inducing apoptosis, tissue damage, organ dysfunction, and possibly DNA damage (6). Thallium binds to sulfhydryl groups in keratins, disrupting keratin cross-linking, which deforms hair, skin, and nails; hallmarks of Tl poisoning (7). The elimination of Tl is primarily via the bile, feces, and urine but it is also excreted in sweat, saliva, breast milk, hair and nails. Intracellular Tl is released from within cells less rapidly than potassium (4). This, combined with the large volume of distribution of Tl, and its extensive enterohepatic recirculation, results in its prolonged half-life in the body (4, 7). The half-life (T_1/2_) in humans is usually between 2 and 4 days, but extended periods of 10–30 days have been documented (6, 7). Experimentally, the T_1/2_ in dogs given 10 mg/kg Tl orally was demonstrated to be 6.5 days (8). This slow excretion rate allows Tl to accumulate in the body, even with low levels of repeated consumption, and traces can be detected months after exposure (4).

In acute Tl intoxications, gastrointestinal symptoms such as vomiting, abdominal pain, diarrhea and/or constipation often occur, within 12 h to 4 days after consumption (1, 7, 9). The gastrointestinal symptoms are typically followed by neurological symptoms (hours to weeks after the exposure) which can range from a painful peripheral neuropathy; presenting as ataxia, hypersensitivity, blindness, dysphonia, and megaoesophagus to severe central nervous symptoms such as delirium, coma and epileptic seizures (1, 3, 7). During this stage, signs of cardiovascular toxicity such as tachycardia and hypertension can be seen (1, 3). Liver and kidney injury are also common in Tl intoxications (1, 10). Dermatological lesions which are considered characteristic for Tl intoxications, including alopecia and dry cracked skin, usually occur two to several weeks after exposure to Tl (1, 7, 10). In severe cases, death has been reported to occur within 1–12 days (4, 5, 12).

The diagnosis of Tl intoxication is based on the observation of exposure, the clinical symptoms, especially dermatological abnormalities, and measurement of Tl in biological samples. For confirmation, Tl can be measured in blood, hair, nails, tissue or urine. Physiological background Tl concentrations in dog hair can range from 1 to 15 mcg/L (3).

The mortality rates range between 70% and 87% in dogs and cats and 6%−15% in humans (3, 5, 11). Early admission of Tl intoxicated patients and the decrease of the toxic load through targeted treatment are associated with a better outcome in humans. The difference in mortalities between humans and animals might be explained by the availability of the antidote/chelator Prussian blue (PB) [potassium ferric-hexacyanoferrate(II)]. Prussian blue exchanges potassium for Tl in the gut thereby reversing the concentration gradient and disrupting the entero-hepatic circulation, resulting in enhanced elimination of Tl (6, 13).

Here, we present a Tl intoxication in a dog successfully treated with PB. Using consecutive serum Tl measurements, we estimate the elimination T_1/2_ of Tl in the presence of PB and compare it to values obtained in an experimental study of Tl elimination in dogs (8).

## Case presentation

A 5-year-old female neutered miniature Schnauzer (8 kg) was taken to the veterinarian due to suspected thallium sulfate (Tl_2_SO_4_) poisoning. The owners lived on a farm and possessed an old bottle of Tl_2_SO_4_ containing rodenticide (Luxan Thalliumsulfaat oplossing 5 g/L, Luxan, Barneveld, The Netherlands). They mixed approximately one quarter of this bottle (1L) with corn and spread the soaked corn in a locked shed 4 days prior to presentation at the veterinary clinic. After the poison had been applied the owner saw that the dog had entered the shed briefly before it was locked, but it had not been seen eating the corn. Over the following days the dog became severely lethargic, anorexic and was frequently regurgitating and corn was found in the feces of the dog. Based on this information, the suspected time of ingestion was 4 days prior to presentation to the veterinarian and the maximum estimated ingested dose of Tl_2_SO_4_ was <1.25 g (<156 mg Tl_2_SO_4_/kg, equivalent to <63 mg TI/kg).

At the veterinary clinic the dog was lethargic, with a temperature of 39.1°C (reference range 38.0–39.0°C), and a tense abdomen, but the rest of the physical exam was unremarkable. A timeline with relevant clinical signs and implemented therapies is outlined in Table 1. Abdominal radiographs were unremarkable but abdominal ultrasonography revealed some echogenic gallbladder sludge, with a thickened pyloric wall and hyperechoic gastric contents. Complete blood count and serum biochemistry were unremarkable except for an increased haematocrit and elevated alanine transaminase (ALT) (Table 2). The Dutch Poisons Information Center was consulted on suspicion of TI poisoning and treatment options were discussed. Prussian blue is not standardly available for veterinary use but the PB (Radiogardase^®^-Cs hard capsule 500 mg, Heyl Chemisch-pharmazeutische Fabrik GmbH & Co. KG, Berlin, Germany) in the national emergency antidote stock for human use was close to expiration, so it was made available for this case. The dog was prescribed 0.25 g/kg of PB per day divided into four doses for 15 days. The dog was also given subcutaneous fluids and prescribed carprofen (Carporal 40 mg, Norbrook Laboratories Limited Newry Co., Down, Northern Ireland) for 10 days and then sent home (Table 1).

**Table 1:** Timeline with relevant clinical signs and therapies including Prussian blue (PB) for 15 days in a 5-year-old, 8 kg, female neutered miniature Schnauzer with thallium sulfate rodenticide poisoning.

**Timeline (days)**	**Clinical status**	**Treatment**
–4 = time of suspected ingestion of thallium		
0 = presentation at veterinary clinic	Body weight: 8 kg	Start of PB therapy: 0.25 g/kg/day PO (divided into 4 doses for 15 days)
	Lethargy, anorexia, regurgitation, tense abdomen	SQ fluids, Carprofen 40 mg (5 mg/kg) PO, once daily for 10 days
3	Worsening lethargy, anorexia, constipation, abdominal pain, borborygmi	Butylscopolamine: 1 mL (2.5 mg/kg) SQ, lactulose: 3 mL (0.25 mg/kg) PO as needed
4	Improved constipation and abdominal pain.	Butylscopolamine: 1 mL (2.5 mg/kg) SQ
5	Body weight: 8.6 kg	Butylscopolamine: 1 mL (2.5 mg/kg) SQ followed by butylscopolamine: 10 mg (1.25 mg/kg) PO twice daily from T = 6 onwards.
	Regained appetite, persistent abdominal discomfort, distended abdomen, increased borborygmi hair loss, mild generalized pustules, serum Tl: 196 mcg/L	
11	Anorexia, lethargy, worsened abdominal discomfort, bald muzzle (Figure 1A), paw licking, scratching and rubbing face	Butylscopolamine: 1 mL SQ
12	Discomfort walking, painful paws, paw nerve pain, paw pad ulceration, nail loss, alopecia, dermatitis, abdominal discomfort	Dexamethasone: 0.5 mL (0.125 mg/kg) SQ, dexamethasone: 1.5 mg (0.18 mg/kg) PO once daily from T = 13 onwards.
14	Walking more comfortably	End of PB therapy
	Serum Tl: 20.7 mcg/L	
20	Eating and drinking independently, walking more comfortably, begin playing, persistent abdominal discomfort	Dexamethasone: weaned over the next 2 weeks, activated charcoal: 33 mL (1 g/kg) three times daily for 4 days
		Vaseline-based cream to be used on paw pads
27	Body weight: 9.2 kg	Vaseline-based cream: continued
	Stable patient, persistent appetite, improved paw pain, persistent cracking paw pads (Figure 1C) and brittle nails, hair regrowth on muzzle	
34	Comfortable on palpation, hair regrowth (Figure 1B)	No further medication
	Serum Tl: 21.5 mcg/L	

SQ, subcutaneous; PO, per os; Prussian blue, PB; Tl, Thallium.

**Table 2:** Hematology and biochemistry results in a 5-year-old, 8 kg, female neutered miniature Schnauzer with thallium sulfate rodenticide poisoning treated with Prussian blue for 15 days, beginning 4 days after suspected ingestion.

**Hematology (units)**	**T = 0 days**	**T = 11 days**	**T = 34 days**	**Reference range**
Red blood cell count (× 10^12/L^)	8.4	7.27	6.49	5.83–9.01
Haematocrit (%)	**61.6**	**55.2**	51.8	36.6–54.5
Hemoglobin (g/dL)	17.2	15.9	14.2	12.2–18.4
Mean cell volume (fL)	**74.8**	**75.9**	**79.8**	55.8–71.6
Mean corpuscular hemoglobin (pg)	20.9	21.9	21.8	17.8–28.8
Mean corpuscular hemoglobin concentration (g/dL)	**27.9**	**28.8**	**27.4**	30.9–38.6
Reticulocytes (K/μL)	81.6	43.0	**138.0**	10.0–110.0
White blood cells count (× 10^9/L^)	8.42	11.61	11.75	5.50–16.90
Neutrophils (× 10^9/L^)	5.87	8.21	9.26	2.00–12.00
Lymphocytes (× 10^9/L^)	1.64	1.50	1.32	0.50–4.90
Monocytes (× 10^9/L^)	0.73	1.74	0.98	0.30–2.00
Eosinophils (× 10^9/L^)	0.15	0.16	0.18	0.10–1.49
Basophils (× 10^9/L^)	0.02	0.01	0.02	0.00–0.10
Platelets (K/μL)	329	364	629	175–500
Glucose (mmol/L)	6.29	5.31	5.84	4.11–7.95
Creatinine (μmol/L)	92	75	48	44–159
Urea (mmol/L)	4.5	6.1	4.6	2.5–9.6
Phosphate (mmol/L)	1.27	1.74	1.76	0.81–2.20
Total calcium (mmol/L)	2.73	2.75	2.72	1.98–3.00
Total protein (g/L)	71	72	75	52–82
Albumin (g/L)	35	33	34	23–40
Globulin (g/L)	35	39	41	25–45
Alanine aminotransferase (U/L)	**387**	**243**	**463**	10–125
Alkaline phosphatase (U/L)	95	111	**552**	23–212
Gamma-glutamyltransferase (U/L)	6	10	**45**	0–11
Total bilirubin (μmol/L)	3	7	7	0–15
Cholesterol (mmol/L)	4.28	5.69	5.45	2.84–8.26
Amylase (U/L)	545	569	554	500–1500
Lipase (U/L)	607	460	627	200–1800
Sodium (mmol/L)	153	-	-	144–160
Potassium (mmol/L)	4.4	-	-	3.5–5.8
Chloride (mmol/L)	117	-	-	109–122

The bold values indicate values which are outside of the indicated reference range

Three days later (T = 3), the owner thought the dog was worsening and becoming more lethargic. The dog accepted force-feeding but did not eat independently. It had produced little feces and had shown discomfort when defecating. Abdominal pain was detected on physical examination with increased borborygmi on abdominal auscultation. The dog was given an injection of 1 mL butylscopolamine (Buscopan 20 mg/mL, Boehringer Ingelheim Spain, Barcelona, Spain) and prescribed oral lactulose to be given until normal feces were produced (Laxatract 667 mg/L, Dechra/AST Farma, Oudewater, the Netherlands). The following day (T = 4) the dog had somewhat improved and had produced a large quantity of feces. The dog was less tense on abdominal palpation and was again injected with 1 mL of butylscopolamine.

On treatment day 6 (T = 5) with PB, the owner reported that the dog was eating and drinking independently. Physical examination showed signs of hair loss. Hair tufts were easily removable without pain, and the dog had mild generalized pustules. The dog had persistent abdominal discomfort, abdominal distension and increased borborygmi. Abdominal radiographs and ultrasound were repeated on which the stomach appeared distended and full of ingesta on both. Ultrasonographically, the pyloric wall seemed less thickened in comparison to the previous week. There was also a cystic structure seen on the spleen, suspected to be an incidental finding. The dog was again injected with butylscopolamine and prescribed twice daily butylscopolamine 10 mg for 30 days (Buscopan, Delpharm Reims, Reims, France).

**Figure 1:**
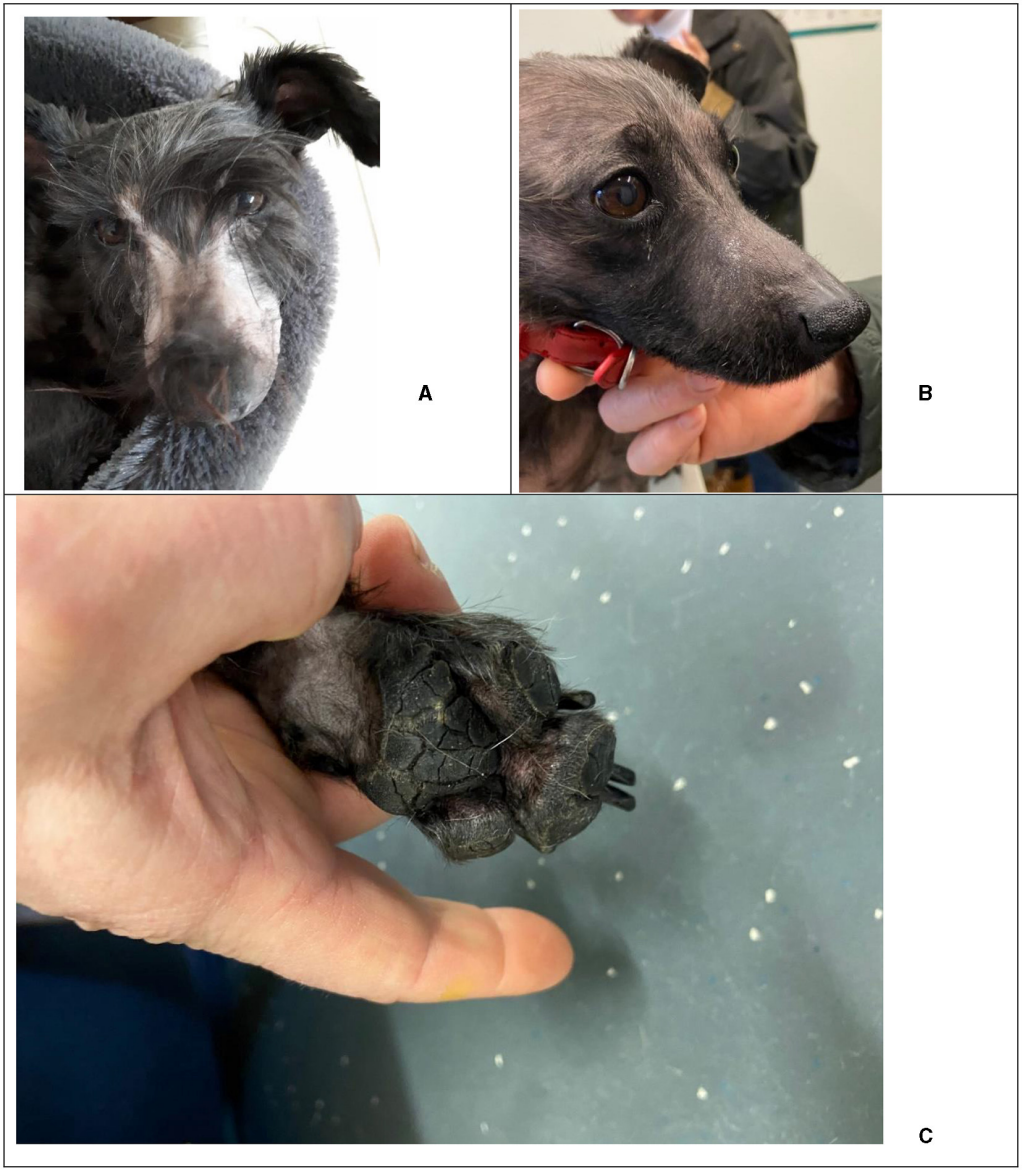
Thallium-induced dermatological effects in a 5-year-old, 8 kg, female neutered miniature Schnauzer: Alopecia on muzzle and head at 11 **(A)** and 34 days **(B)**, and cracked paw pad **(C)** 27 days after the start of Prussian blue treatment.

Six days later (T = 11), the owner reported that the dog had deteriorated. The dog was lethargic, again anorexic and requiring force-feeding, and had become bald in the muzzle region (Figure 1A). At home, the dog was scratching and rubbing its eyes and muzzle region and constantly licking its paws which seemed to be painful. On physical examination the abdomen was again tense on palpation. On blood examination the haematocrit and ALT were less elevated compared to day 0 (Table 2). The dog was again injected with butylscopolamine.

The following day (T = 12), the dog had discomfort walking and was painful by paw palpation, which the veterinarian interpreted as “nerve pain.” Her paw pads were beginning to crack and her nails were falling out. The dog had generalized alopecia with a moderate dermatitis and was still uncomfortable on abdominal palpation, so a dexamethasone injection (Rapidexon 2 mg/mL, Eurovet Animal Health BV, Bladel, the Netherlands) 0.5 mL was given, and was prescribed oral dexamethasone (Dexacortone 0.5 mg, Dechra/AST Farma, Oudewater, the Netherlands) 1.5 mg once daily. A few days later (T = 14), the owner reported that the dog seemed to be doing well on the medication and was walking more comfortably. On this day the PB therapy was ended.

On day 20, the dog was eating and drinking independently, walking comfortably, and playing again. The oral dexamethasone was weaned downwards over the following 2 weeks. The dog continued to have abdominal discomfort on physical examination. As Tl was still detected in serum on T = 14, activated charcoal (1 g/kg, three times daily) was prescribed to enhance elimination (Carbodote 240 g/L; TVM UK Animal Health Ltd, Oxfordshire, United Kingdom) for 4 days. The owners were also advised to apply a vaseline-based skin cream to the paws multiple times daily (Purol; Labori International BV, Minervum, Breda, the Netherlands).

On day 27, the owner reported that the dog remained stable and was eating and drinking well. It still seemed to have painful paws and the paw pads and nails were still cracking (Figure 1C) but the owners felt the cream helped the dog become more comfortable. Hair had begun to return on the muzzle of the dog. On day 34, the final day of medical management, the dog was more comfortable on abdominal palpation and hair was continuing to re-grow (Figure 1B). On the final blood examination (Table 2) the dog had a normal haematocrit, an elevated ALT, alkaline phosphatase (ALP) and gamma-glutamyl transferase (GGT).

During telephone consultation 3 years later, the owners reported that the dog continued to have some alopecia and discomfort in the paws until ~6 weeks after the Tl ingestion and appeared fully recovered by 11 weeks.

### Thallium concentrations and toxicokinetics

To confirm the suspicion of Tl intoxication, blood samples were analyzed by ICP-MS (inductively coupled plasma-mass spectrometry, IDEXX laboratories, Hoofddorp, The Netherlands). The serum concentration was 196 mcg/L 9 days after the suspected exposure. This decreased to 20.7 mcg/L at the end of the PB treatment and 21.5 mcg/L 38 days after the suspected exposure. This slightly increased last measurement 11 days after finishing targeted therapy (PB followed by activated charcoal) is difficult to explain, but might be due to reaching a plateau phase or a measurement error.

**Figure 2:**
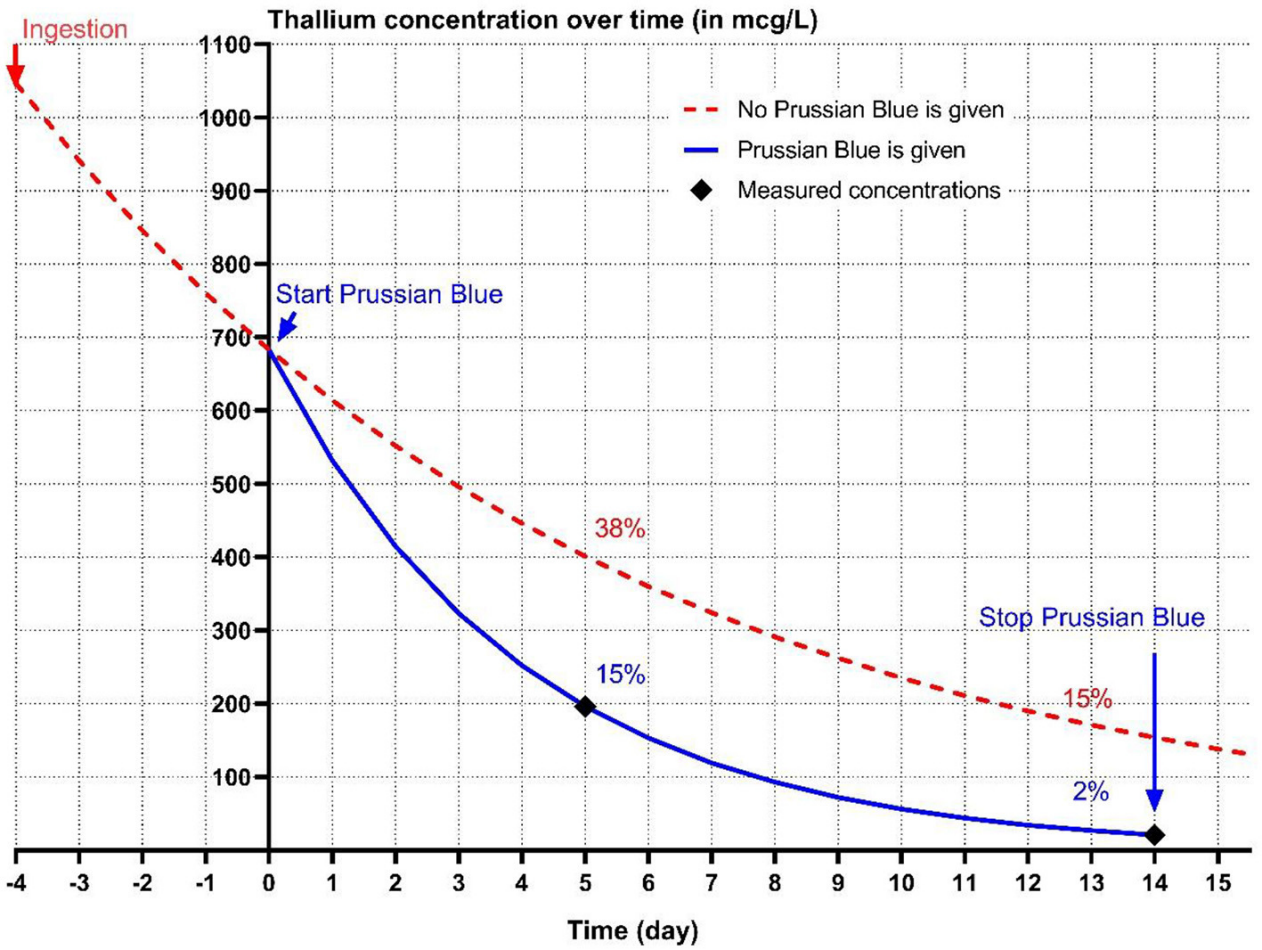
The conjectured kinetics of thallium (Tl) excretion without (red dotted line) Prussian blue (PB) treatment based on experimental data from Van der Stock et al. (8). The blue line represents the Tl concentration based on the estimated half-life (2.8 days) of Tl during PB treatment in a 5-year-old, 8 kg, female neutered miniature Schnauzer treated with PB course of 15 days, 4 days after suspected ingestion of Tl. Measured Tl concentrations on T = 5 and T = 14 (day 9 and day 18 after suspected ingestion, respectively), are indicated by a black diamond.

A T_1/2_ of 2.8 days was calculated during PB treatment based on the first two measurements of Tl, assuming first order kinetics, and using the exponential decay formula ($C_{t}=C_{0}{\left(\frac{1}{2}\right)}^{\frac{t}{t_{1/2}}}$). The initial serum Tl concentration was estimated to be 1046.6 mcg/L, using an elimination half-life of 6.5 days from the day of exposure till start PB treatment and 2.8 days during PB treatment (8). In Figure 2, the effect of continuous PB treatment from day 5–20 after ingestion on the total body Tl load in this real case (blue line) was plotted against Van der Stock et al.'s experimental data on dogs given Tl without PB (8) (red dotted line). We estimate that PB reduced Tl levels to 15% on day 5, compared to 38% without PB, and to 2% on day 14, vs. 15% without PB (Figure 2).

## Discussion

The clinical signs (gastrointestinal, neurological and dermatological) in this case are consistent with the literature relating to Tl intoxications. Despite a high maximum ingested dose, symptoms frequently seen in fatal cases, such as cardiovascular abnormalities, convulsions, coma and severe mental depression, were absent (14). This could be related to the use of PB and/or suggest that the dog consumed a lesser dose of Tl.

The gastrointestinal symptoms in the dog consisted of initial regurgitation, borborygmi, constipation, prolonged intermittent anorexia and abdominal discomfort. Early symptoms of acute Tl intoxication in dogs often consist of nausea, vomiting, anorexia, abdominal pain, and diarrhea which can be haemorrhagic in severe cases due to a necrotizing gastroenteritis (4, 10). The lack of more severe gastrointestinal symptoms such as vomiting and diarrhea in this case is interesting and could be due to the fact that the dog was suffering from a milder gastroenteritis visualized as the thickened pylorus seen on the abdominal ultrasounds. The initial regurgitation could have been related to esophageal reflux or dysmotility however there were no thoracic radiographs performed to confirm this. The abdominal discomfort and anorexia were likely initially due to Tl induced gastroenteritis and later due to constipation/dysmotility caused by Tl or PB treatment. The constipation in Tl intoxications is suggested to be due to depressed intestinal motility and peristalsis, related to direct vagus nerve involvement and impairment of the autonomic nervous system (ANS), more often seen later in the clinical course (15–17). However, the presence of both increased borborygmi and constipation causing abdominal discomfort and pain in this case are more suggestive of increased, ineffective peristalsis in this dog. The PB may have mitigated late ANS damage in this case, given the relatively mild clinical signs and short constipation duration. Treatment of the abdominal pain in this dog proved challenging and butylscopolamine seemed to be effective. As a parasympatholytic, it is possible butylscopolamine relieved discomfort related to direct vagus nerve involvement, such as excessive borborygmi and intestinal cramping. Many analgesics (e.g., opiates, paracetamol, non-steroidal anti-inflammatories) pose additional risks in Tl intoxication patients, especially those with impaired intestinal motility, liver dysfunction or those at-risk of GI ulceration.

The neurological signs in this patient are also suggestive of a milder intoxication and/or the positive effect of the PB. Consistent with Tl poisoning, the dog showed suspected vagal nerve abnormalities followed by painful peripheral neuropathy in the 2nd week, though more severe symptoms like ataxia, tremors, and coma never developed (15). Furthermore, the dog had no chronic neurological defects such as tremors, ataxia and megaoesophagus, which are seen in some Tl intoxication survivors (3, 14). As to whether the regurgitation was a temporary neurological symptom is unclear, but dogs can develop a megaoesophagus as part of the peripheral neuropathy believed to be related to a diffuse axonopathy in a Wallerian degeneration pattern, possibly involving the nerves which regulate esophageal motility (3, 17). The dog displayed constipation which also could have been a neurologic or GI symptom which may have been relieved through the use of mannitol, cisapride or lactulose (17–19). The persistent borborygmi indicates impaired peristalsis rather than complete paralytic ileus from ANS dysfunction, which could have developed without the PB use. The painful paws and painful walking are very fitting of Tl intoxication. Despite the coinciding onset of dermatological symptoms, the dog's paw licking before the development of cracked pads suggest the pain was at least in part neurological, rather than solely dermatological.

Skin lesions are visible in the later stages of Tl intoxication and are related to the interference of Tl in keratin cross-linking. Alopecia, although not always present, is the best-known dermatological symptom of Tl intoxication, and it tends to start in areas of cutaneous friction (1, 15). Alopecia, begins around the lips, nasal cleft, and ear margins, spreading to the face, head, and abdomen, often sparing portions of the eyebrows, as seen in this dog (Figures 1A, C) (14, 15, 20). Other typical dermatological symptoms including secondary dermatitis, pyoderma, hyperkeratosis, cracking/ulcerations of the footpads and brittle nails were also displayed in the dog (Figure 1B) (1, 4). Conjunctivitis and mahogany-colored mucous membranes, common in Tl intoxications, were absent in this dog (1).

The dog's haemoconcentration at presentation, followed by hyperchromic and macrocytic red cells after normalization of haematocrit, may indicate a measurement error, and/or initial dehydration which was subsequently corrected or a haemolytic anemia, sometimes reported in Tl toxicity (15). Worsening of liver values (ALT, ALKP and GGT) of unknown clinical relevance, was also seen, which is in keeping with human literature where signs of continuing liver damage can be present for a longer period of time (6).

The initial estimated maximum ingested dose (<63 mg Tl/kg) greatly exceeds the minimal lethal dose in dogs of 10–15 mg/kg (1, 21). This estimate was based on a “worst case scenario” in which the dog ingested all the Tl soaked corn. Though the owner indicated that not all the corn was eaten, we do not know the exact ingested dose. The ingested dose can be roughly estimated from the calculated initial serum Tl concentration (1,046.6 mcg/L) multiplied by the volume of distribution [average Vd 3.6 L/kg (13)], resulting in a total thallium body load of ~3.8 mg/kg, which is below the reported minimal lethal dose for dogs. An earlier report describes a dog exposed to ~3.9 mg Tl/kg with a blood Tl concentration of 190 mcg/L 19 days after exposure. This dog survived without PB treatment but suffered esophageal paralysis and megaoesophagus for 10 months (3). Another dog survived a dose of 3.7 mg Tl/kg, although it developed vomiting and bloody diarrhea, hair loss 7 days post-ingestion and was released from the clinic 43 days after exposure (3, 4). The dog in the present case survived an estimated oral dose of ~3.8 mg Tl/kg, was treated at home and fully recovered by 11 weeks probably due to the PB induced enhanced elimination of Tl.

As Tl undergoes extensive entero-hepatic circulation, the optimal treatment of Tl intoxications involves supportive therapy and reducing the toxic load by disturbing the entero-hepatic circulation (17, 22, 23). Prussian blue and to a lesser extent activated charcoal, absorb Tl cations in the GI tract and prevent their re-absorption from the GI tract thereby enhancing their excretion in the feces (13, 17). Stock et al. report a T_1/2_ of 6.5 days of Tl in dogs without the presence of PB, which subsequently reduced to 2.4 days when they were treated with PB. We could not calculate the T_1/2_ without PB treatment, but using the first two serum Tl measurements, we calculated a T_1/2_ of 2.8 days during PB treatment, a value very similar to that obtained in the above experimental study (8).

In humans PB and charcoal are frequently given at the beginning of treatment, or charcoal is given while waiting for PB and discontinued once PB can be given (17). Prussian blue is usually administered for ~30 days and/or until Tl levels return to a safe range (19). Safe Tl levels in dogs range from 1 to 15 mcg/L, which the dog in the present study still exceeded at the end of its PB therapy (3). Therefore, a 4 day course of activated charcoal was administered. Extracorporeal therapy alone is likely to be insufficient for patients with Tl poisoning and it should be an addition to the above outlined treatments (17). A multidisciplinary consensus group advocates the use of a combination of haemodialysis and haemoperfusion in case of severe Tl poisoning, ideally initiated within 24–48 h after ingestion while serum Tl concentrations are high (7, 17).

The current case of Tl poisoning demonstrates that although widely unavailable, Tl intoxications can still occur. Thallium intoxication should be considered as a differential diagnosis when a triad of gastrointestinal, neurological and dermatological symptoms occur. The preferred targeted therapy for Tl intoxications is Prussian blue, which is rarely available or used in veterinary settings. The calculated elimination half-life, roughly estimated body load of Tl and the dog's relatively mild symptoms, suggests the use of PB contributed to the quick recovery of the dog compared to similar cases.

## Data Availability

The original contributions presented in the study are included in the article, further inquiries can be directed to the corresponding author.

## References

[1] Skelley JF Gabriel KL. Thallium intoxication in the dog. Ann N Y Acad Sci. (1964) 111:612–7. 10.1111/j.1749-6632.1964.tb53129.x 14172735

[2] Thompson LJ. Chapter 14–thallium. In:Gupta RC, editor. Handbook of Toxicology of Chemical Warfare Agents. 2nd ed. Boston, MA: Academic Press (2015). p. 167–70. 10.1016/B978-0-12-800159-2.00014-2

[3] Puschner B Basso MM Graham TW. Thallium toxicosis in a dog consequent to ingestion of mycoplasma agar plates. J Vet Diagn Invest. (2012) 24:227–30. 10.1177/1040638711425941 22362959

[4] Volmer PA Merola V Osborne T Bailey KL Meerdink G. Thallium toxicosis in a pit bull terrier. J Vet Diagn Invest. (2006) 18:134–7. 10.1177/104063870601800124 16566274

[5] Nunes Rodrigues TC Mortier F De Baets J Vandenabeele SI. Thallium toxicosis in a brittany spaniel. Vet Rec Case Rep. (2021) 9:e166. 10.1002/vrc2.166

[6] Fujihara J Nishimoto N. Thallium-poisoner's poison: an overview and review of current knowledge on the toxicological effects and mechanisms. Curr Res Toxicol. (2024) 6:100157. 10.1016/j.crtox.2024.100157 38420185 PMC10899033

[7] Ghannoum M Nolin TD Goldfarb DS Roberts DM Mactier R Mowry JB. Extracorporeal treatment for thallium poisoning: recommendations from the EXTRIP workgroup. Clin J Am Soc Nephrol. (2012) 7:1682–90. 10.2215/CJN.01940212 22837270

[8] Van der Stock J De Schepper J. The effect of Prussian blue and sodium-ethylenediaminetetraacetic acid on the faecal and urinary elimination of thallium by the dog. Res Vet Sci. (1978) 25:337–42. 10.1016/S0034-5288(18)32950-3 107548

[9] Al Hammouri F Darwazeh G Said A Ghosh RA. Acute thallium poisoning: series of ten cases. J Med Toxicol. (2011) 7:306–11. 10.1007/s13181-011-0165-3 21735311 PMC3550187

[10] Zook BC Gilmore CE. Thallium poisoning in dogs. J Am Vet Med Assoc. (1967) 151:206–17. 6068222

[11] Zou H Zou S. Advanced thallium toxicity. Pract Neurol. (2022) 2022:1–11. 10.1155/2022/7266037 36424143

[12] Lennox WJ. Thallium poisoning. Can Vet J. (1966) 7:113. 5948852 PMC1696404

[13] Hoffman RS. Prussian blue. In:Nelson LS Howland MA Lewin NA Smith SW Goldfrank LR Hoffman RS, editors. Goldfrank's Toxicologic Emergencies. 11th ed. New York, NY: McGraw-Hill Education (2019). p. 1357–61.

[14] Reed D Crawley J Faro SN Pieper SJ Kurland LT. Thallotoxicosis: acute manifestations and sequelae. JAMA. (1963) 183:516–22. 10.1001/jama.1963.03700070044007 13973536

[15] Mulkey JP Oehme FW A. review of thallium toxicity. Vet Hum Toxicol. (1993) 35:445–53. 8249271

[16] Galván-Arzate S Santamaría A. Thallium toxicity. Toxicol Lett. (1998) 99:1–13. 10.1016/S0378-4274(98)00126-X 9801025

[17] Zappala MM Hoffman RS. Thallium. In:Nelson LS Hoffman RS Howland MA Lewin NA Goldfrank LR, editors. Goldfrank's Toxicologic Emergencies. 11th ed. New York, NY: McGraw-Hill Education (2019). p.1350–6.

[18] Vrij AA Cremers H Lustermans FT. Successful recovery of a patient with thallium poisoning. Neth J Med. (1995) 47:121–6. 10.1016/0300-2977(95)00006-9 7566291

[19] Hoffman RS. Thallium toxicity and the role of Prussian blue in therapy. Toxicol Rev. (2003) 22:29–40. 10.2165/00139709-200322010-00004 14579545

[20] Gwaltney-Brant SM. Chapter 41–heavy metals. In:Haschek WM Rousseaux CG Wallig MA, editors. Haschek and Rousseaux's Handbook of Toxicologic Pathology. 3rd ed. Boston, MA: Academic Press (2013). p. 1315–47.

[21] Waters CB Hawkins EC Knapp DW. Acute thallium toxicosis in a dog. J Am Vet Med Assoc. (1992) 201:883–5. 10.2460/javma.1992.201.06.883 1399797

[22] Riyaz R Pandalai SL Schwartz M Kazzi ZN. A fatal case of thallium toxicity: challenges in management. J Med Toxicol. (2013) 9:75–8. 10.1007/s13181-012-0251-1 22865288 PMC3576490

[23] Wallbridge T James S Lee R Khan A Bradberry S Elamin ME. Successful treatment of potentially lethal dose thallium sulfate poisoning with sequential use of Prussian blue and multiple-dose activated charcoal. Clin Toxicol. (2023) 61:200–1. 10.1080/15563650.2023.2165502 36794304

